# Demographic changes and marker properties affect detection of human population differentiation

**DOI:** 10.1186/1471-2156-8-21

**Published:** 2007-05-11

**Authors:** Jennifer B Listman, Robert T Malison, Atapol Sughondhabirom, Bao-Zhu Yang, Ryan L Raaum, Nuntika Thavichachart, Kittipong Sanichwankul, Henry R Kranzler, Sookjaroen Tangwonchai, Apiwat Mutirangura, Todd R Disotell, Joel Gelernter

**Affiliations:** 1 Dept Anthropology, New York Univ, NY, USA; 2 Dept Psychiatry, Yale Univ Sch Medicine, New Haven, CT, USA; 3 VA CT, West Haven, CT, USA; 4 Chulalongkorn Faculty of Med, Bangkok, Thailand; 5 Dept Psychiatry, Univ of CT Sch Medicine, Farmington, CT, USA; 6 Dept Anthropology, Univ of FL, Gainesville, FL, USA; 7 Suan Prung Psychiatric Hospital, Chiang Mai, Thailand; 8 Depts Genetics and Neurobiology, Yale Univ Sch Medicine, New Haven, CT, USA

## Abstract

**Background:**

Differentiating genetically between populations is valuable for admixture and population stratification detection and in understanding population history. This is easy to achieve for major continental populations, but not for closely related populations. It has been claimed that a large marker panel is necessary to reliably distinguish populations within a continent. We investigated whether empirical genetic differentiation could be accomplished efficiently among three Asian populations (Hmong, Thai, and Chinese) using a small set of highly variable markers (15 tetranucleotide and 17 dinucleotide repeats).

**Results:**

Hmong could be differentiated from Thai and Chinese based on multi-locus genotypes, but Thai and Chinese were indistinguishable from each other. We found significant evidence for a recent population bottleneck followed by expansion in the Hmong that was not present in the Thai or Chinese. Tetranucleotide repeats were less useful than dinucleotide repeat markers in distinguishing between major continental populations (Asian, European, and African) while both successfully distinguished Hmong from Thai and Chinese.

**Conclusion:**

Demographic history contributes significantly to robust detection of intracontinental population structure. Populations having experienced a rapid size reduction may be reliably distinguished as a result of a genetic drift -driven redistribution of population allele frequencies. Tetranucleotide markers, which differ from dinucleotide markers in mutation mechanism and rate, are similar in information content to dinucleotide markers in this situation. These factors should be considered when identifying populations suitable for gene mapping studies and when interpreting interpopulation relationships based on microsatellite markers.

## Background

Genetic characterization and differentiation of populations are often necessary for the conduct of valid case-control association studies [1-5], determining the role of ancestry in phenotypic differences [6,7], assigning population groups for valid linkage analysis [8], examining the distribution of neutral genetic variation among populations, and inferring migration histories [9-11]. Such differentiation has been accomplished with relative ease between major continental populations [10,12-15], but it has been asserted that population differentiation *within *a continent may not be possible; and when it appears to be so, may actually be an artifact of study design [16].

The ubiquity and frequently highly variable nature of short tandem repeat polymorphisms (STRs or microsatellites) have made them desirable markers for measuring population stratification. Commercially available marker sets such as those used for forensic purposes make STR genotyping cost effective, eliminating the time and effort required to develop multiplex marker panels. Panels developed for forensic purposes are designed to identify or exclude an individual as a match for another sample and were compiled, in part, for their high levels of variation in many populations [17]. Such panels have been adopted for non-forensic purposes such as inference of population phylogenies [18-21] and quantification of levels of population differentiation [1,2,5,8].

Homoplasy, as applied to STRs, refers to the situation where alleles of the same length have arisen from different mutation events, such that alleles identical-by-state are not necessarily identical-by-descent. Simulations of STR evolution using the stepwise mutation model (SMM) have indicated that homoplasy in STR genotypes may cause individuals or populations to appear to be more genetically similar than they really are. Point mutations, insertion or deletion events (indels), or complex repeat motifs can generate additional forms of size homoplasy that are sometimes revealed by sequencing but are not detectible through size fractionation (electrophoresis) [22-24]. These forms of homoplasious alleles have been observed in a number of the tetranucleotide repeats that are standard in forensic panels (some of which are included among the markers used in this study; see below) [25].

However, it has also been shown that even in the presence of homoplasy, multi-locus genotypes (the combined genotypes from multiple loci) of highly variable STR markers are effective in assigning individuals to known or unknown populations [26-33]. Again, this has typically been true for large continental populations. Population differentiation *within *a continent has been successful, but only with large numbers of markers when applied to population isolates [10]. Here, we used a small set of markers and, in contrast to the majority of past studies, addressed the properties of the markers used.

Conditions such as small population size or recent founding of a population may enable statistical differentiation using a small panel of highly variable markers, due to increased effects of genetic drift and decreased incidence of homoplasy. To evaluate this possibility, we investigated whether empirical genetic differentiation could be accomplished efficiently among three closely related Asian populations (Hmong, Thai, and Chinese) using a small set of STRs that includes both tetranucleotide and dinucleotide markers. In addition, we studied the relative information content of tetranucleotide versus dinucleotide markers for discriminating among these three Asian populations, as well as European Americans (EA) and African Americans (AA). We then evaluated the populations for evidence of recent changes in effective population size.

## Results

### Population differentiation

**Figure 1:**
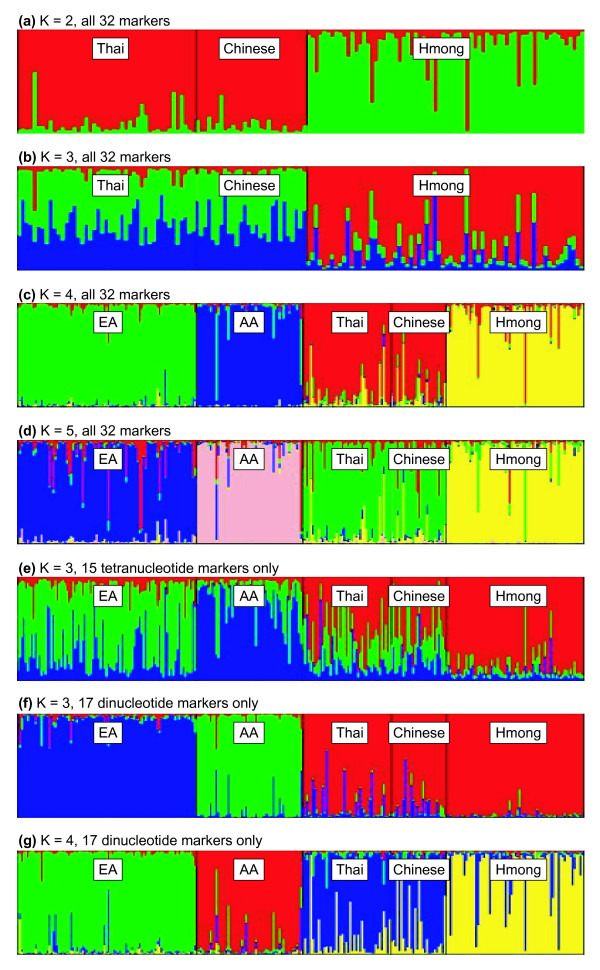
Hmong populations are consistently and reliably distinguished from all others in STRUCTURE analyses using a small number of either dinucleotide or tetranucleotide markers while for other populations successful assignment of individuals varies with marker type. In **(a) **and **(b) **Chinese, Hmong, and Thai samples were assigned by STRUCTURE to 2 or 3 populations respectively. In **(c) **and **(d) **European-American (EA), African-American (AA), Chinese, Hmong and Thai samples were assigned to 4 or 5 populations respectively. Finally, tetranucleotide markers **(e) **are less useful for differentiating among EA, AA, Thai and Chinese populations than dinucleotide markers **(f & g)**. These plots were produced using the STRUCTURE software; each individual is represented by a vertical line depicting the estimated percent assignment of the individual into K assumed populations. Each assumed population is represented by one color. Vertical black lines separate individuals by self-reported ancestral population.

The program STRUCTURE 2.1 [32,33] uses Bayesian clustering of multilocus genotypes to assign individuals to populations, estimate admixture proportions for individuals, and infer the number of parental populations (K) for a sample. For STRUCTURE runs which included the three East Asian populations only and all 32 markers, the Hmong were allocated into a cluster distinct from a single Thai/Chinese cluster with 86.0% estimated ancestry for K = 2 with a posterior probability (Pr(K = 2)) of 1, indicating K with the best fit for the data (Figure 1a). Separate Thai and Chinese clusters were not inferred with K = 3 and Pr(K = 3) was effectively zero (3.3 × 10 ^-156^) (Figure 1b). When the three East Asian populations were analyzed with EA and AA samples, the Hmong were then allocated to a separate cluster with an average of 90.0% estimated ancestry when K = 4 and Pr(K = 4) = 1 (Figure 1c). Under these same conditions, the Thai and Chinese were assigned together to a single cluster with 86.5% and 84.2% estimated ancestry, respectively. When K was increased to 5, the Thai and Chinese populations continued to form a single cluster (Figure 1d) and Pr(K = 5) was 1.7 × 10^-48^. For K = 2, K = 3, or K = 6, Pr(K) was similarly effectively zero (barplots not shown).

When the markers were separated by repeat size the extent of successful population assignment differed greatly between the two panels; for STRUCTURE runs using the 15 tetranucleotide markers alone Pr(K = 3) was 1, while for the 17 dinucleotide markers alone Pr(K = 4) was 1. In addition, although K = 3 had the best fit for the data for tetranucleotide markers, assignment of individuals to major continental populations was not robust (Figure 1e) (EA 63.2%, AA 75.3%, Thai 54.1%, Chinese 48.7%, Hmong 86.1%). Dinucleotide markers alone resulted in higher assignment rates than those of the tetranucleotide markers when K = 3 (Figure 1f) (EA 94.0%, AA 91.4%, Thai 88.3%, Chinese 88.4%, Hmong 97.3%) or under the best fit for the data, K = 4 (Figure 1g) (EA 91.4%, AA 90.4%, Thai 81.0%, Chinese 73.6%, Hmong 82.8%).

Out of concern that each Hmong village in which samples were collected could consist of its own apparent cluster due to close relatedness within each village, the villages were analyzed initially as separate populations in STRUCTURE under the same conditions as all STRUCTURE runs reported here. In all cases, the two source villages formed one cluster and the average assignment values for all population samples, including Hmong, were no different than assignment values reported here when the two villages were combined and assumed to be one population (data not shown).

### Effective population size

The Hmong sample was found to have a heterozygosity deficiency (p = 0.004), based on a sign test in BOTTLENECK [34], indicating a possible recent population expansion. Given the number of observed alleles, if the Hmong population was at equilibrium heterozygosity is expected to be higher than that which is observed. All other samples had neither excess nor deficiency for this measure.

### Relatedness

Based on maximum-likelihood estimates of pair-wise relationships, potential parent-offspring pairs and sibling pairs were discovered in the Chinese and Hmong samples. In each case, one individual was then deleted from the sample and excluded from all other analyses.

### Hardy Weinberg Equilibrium (HWE)

No population showed significant deviation from HWE over all loci (EA p = 0.07, AA p = 0.82, Chinese p = 0.57, Thai p = 0.87, Hmong p = 0.99) (Table 1). If a Bonferroni correction is applied to correct for multiple testing, (requiring a p value of 0.05/32 = 0.00156 for significance) none of these p-values for individual loci are significant (Additional File 1).

**Table 1:** Results from Fisher's test for deviation from HWE for all 32 loci combined

	**EA**	**AA**	**Thai**	**Chinese**	**Hmong**
chi square	81.3	53.6	51.5	61.3	**41.6**
df	64	64	64	64	64
p-value	0.07	0.82	0.87	0.57	0.99

### Heterozygosity

The mean observed heterozygosity (H_o_) (Table 2) for all loci was not statistically different for any of the Asian population pairs, based on paired two-sample t-test (Chinese/Hmong p = 0.07, Thai/Chinese p = 0.34, Thai/Hmong p = 0.27). With the exception of EA/Chinese, mean observed heterozygosity was significantly different for all other population pairs (EA/AA p = 0.01, EA/Thai p = 0.03, EA/Chinese p = 0.37, EA/Hmong p < 0.01, AA/Hmong p < 0.01, AA/Thai p < 0.01, AA/Chinese p = 0.04).

**Table 2:** Mean Nei's gene diversity (H_z_)and mean observed heterozygosity (H_o_)for all markers for each population

	**N**	**H_z_**	**H_z _SD**	**H_o_**	**H_o _SD**
EA	91	0.77	0.01	0.76	0.01
AA	54	0.81	0.01	0.81	0.01
Thai	45	0.74	0.02	0.72	0.01
Chinese	28	0.75	0.03	0.74	0.01
Hmong	70	0.71	0.02	0.69	0.01

Mean tetranucleotide H_o _was not significantly different from mean dinucleotide H_o _for any population other than Chinese based on a two-sample t-test (Table 3) (AA p = 0.49, EA p = 0.17, Thai p = 0.30, Chinese p = 0.05, Hmong p = 0.57).

**Table 3:** Mean observed heterozygosity (H_o_) for each marker type for each population

	**EA**	**AA**	**Thai**	**Chinese**	**Hmong**
H_o _tetranucleotide	0.78	0.80	0.74	0.80	0.71
H_o _dinucleotide	0.75	0.82	0.69	0.68	0.68

### Marker information content

**Figure 2:**
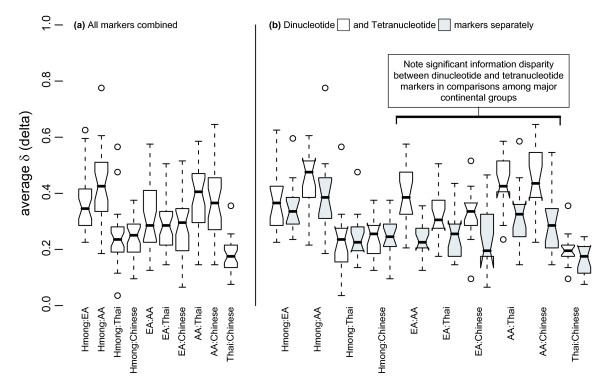
The left side of the figure shows the average delta for all 32 loci for each population pair and the right side of the figure shows the average delta for 15 tetranucleotide (shaded) and 17 dinucleotide (unshaded) markers, separately, for each population pair.

The mean Hmong/Thai and Hmong/Chinese δ values are nearly equivalent, and the Hmong were similarly differentiated from these two populations (delta values for each locus and mean delta values for all loci and by repeat size are reported for each population pair in Additional File 2). The low mean Chinese/Thai δ appears to explain the inability of this marker panel to assign the Thai and Chinese to separate clusters. Overall, the dinucleotide markers provide more information than the tetranucleotide markers, but this difference is not as great for population pairs that include the Hmong; for the Hmong/Chinese and Hmong/Thai population pairs, the difference in the average dinucleotide δ and the average tetranucleotide δ is negligible (Figure 2).

## Discussion

In this study, we successfully differentiated between closely related populations using a marker set much smaller than that previously suggested to be minimally necessary for such studies. We used a set of highly polymorphic microsatellite markers of which some were specifically selected for high δ between EA, AA, and Asian populations [15], however, the value of this marker set for differentiating populations within Asia was previously unknown. To explain our results, we investigated the evolutionary histories of the samples, and found evidence for changes in N_e _for the Hmong population, based on an excess of rare alleles. This tribal population has a recent history of repeated fractioning and migration throughout Southeast Asia as well as loss of numbers due to military conflict, which is consistent with our results [35]. Further suggestive evidence of a recent Hmong population bottleneck followed by expansion can be found in the delta values of tetranucleotide markers compared to that of dinucleotide markers. Delta measures absolute values of allele frequency differences which can arise over time via accumulated mutations or through deviations from neutral conditions such as drift caused by a bottleneck.

If time since divergence determines differences in allele frequencies delta should be correlated with time since divergence. The tetranucleotide markers consistently provide as much information for the Hmong as the dinucleotide markers provide, while this is not so for any other population. We propose that this suggests forces other than mutation as measured by divergence time contributing to differences in population allele frequencies between Hmong and other populations examined here. The effects of this can be seen in the differences in clustering behavior using STRUCTURE when either tetranucleotide or dinucleotide genotypes are analyzed alone – specifically, the difficulty in using the tetranucleotide panel to assign individuals to major continental groups for all populations, with the exception of the Hmong. Rosenberg et al [30] found dinucleotide markers to be more informative than tetranucleotide markers for population assignment in a larger study based on a different measure of marker informativeness. In their study, populations from the Americas or Oceania were exceptions to this pattern. The authors proposed genetic drift during founding events as one explanation for their results.

Mean tetranucleotide H_o _was not statistically different from mean dinucleotide H_o _for any population other than Chinese. Although these markers suggest high intrapopulation variation for all populations in this study, this does not provide information on differences in the sources of that variation either within or between populations for each type of marker.

The decrease in difference between δ values between the two subsets of markers for population combinations that include the Hmong indicates that genetic drift (random changes in allele frequencies from one generation to the next that are more likely to affect small populations) rather than mutation has been a major force of evolution contributing to observed allele frequencies in this population. Differences in marker information content between the tetranucleotide and dinucleotide panels for all other populations in this study indicate that mutation rate and mechanism have shaped allele frequency distributions in these populations more than genetic drift, as would be expected for large populations at mutation-drift equilibrium.

The dinucleotide markers were previously selected for differentiation between European and African populations and high variation [15] and the tetranucleotide markers were chosen for forensic purposes for their high rates of variation in multiple populations.

Total sample size, unequal sample size between populations, and number of markers can affect the stability of clustering in STRUCTURE [36,37]. We cannot exclude biases introduced through these study design elements influencing our observations, however, stable clustering patterns were inferred in this case by repeated STRUCTURE runs. Although increasing the number of markers or population sample size can strengthen clustering patterns where clustering exists, the number of individuals in a sample or the minimum number of markers necessary to differentiate between all populations is dependant on the evolutionary histories of the population samples. Sample sizes similar to ours have been demonstrated previously to be generally sufficient for stable and accurate clustering [36].

Some of the tetranucleotide markers in this study have been shown to consist of complex repeats including more than one repeat motif, as well as insertions or deletions of partial repeats [17] (structure of observed alleles and their amplicon sizes can be found for tetranucleotide repeats typically used for forensic purposes and in this study at [25]. These factors, as well as historically large effective population size such as those of the EA and AA populations, increase the likelihood of size homoplasy. We hypothesize that the accumulation of homoplasious alleles of tetranucleotide loci may contribute to their lower information content when compared to that of the dinucleotide markers in populations other than Hmong.

## Conclusion

When STR loci are used either to detect association, linkage, or population substructure, population history and marker choice both affect study results. Demographic history and marker properties are often overlooked when determining population or marker suitability for gene mapping studies (i.e. to identify variants that affect traits), but have bearing on the efficiency and feasibility of such studies.

The three Asian populations in this study have mean H_o _values which are not significantly different from each other. It is likely that genetic drift, in conjunction with long-standing endogamy, allow the Hmong to be statistically differentiated from the Thai and Chinese using multilocus genotypes, despite the high level of within-population variation of the Hmong. Potential homoplasy in populations at equilibrium warn against the use of STRs (particularly those with known homoplasious alleles) for phylogenetic analysis or linkage or association mapping purposes in large populations, other than quantifying population stratification. Since the tetranucleotide repeats used in commercially available kits designed for forensic purposes have been well-characterized and have been demonstrated to contain many instances of size homoplasy, these markers should not be relied upon for phylogenetic analyses. Risks of homoplasy interfering with association or linkage analysis, in which identical-by-state is often assumed to mean identical-by-descent, should be less of a concern in populations that have undergone recent bottlenecks.

In addition, a history of migrations or bottlenecks in an isolated population is expected to initially reduce levels of intrapopulation variation, and increase interpopulation differentiation [38]. Linkage disequilibrium (LD) will be higher in such populations [39,40]. A subsequent population expansion will recover allelic variation faster than LD will degrade for a given genomic region. Low intrapopulation variation, the corresponding increase in interpopulation variation, and higher LD, have been identified as desirable characteristics in a population for gene mapping and admixture detection [41,42], making geographically or culturally isolated populations with a history of bottlenecks potentially more valuable for gene mapping than populations whose size has remained stable and large. In addition to reducing genetic heterogeneity for the trait of interest, use of such populations also could reduce the costs of association mapping studies because the number of subjects needed for a specified power level is inversely related to the population's level of LD [43]. It would therefore be useful to identify such populations prior to designing a study.

Existence of a large number of rare alleles within a population can be the result of a bottleneck followed by expansion [44-46]. It is possible to infer these events from a significant heterozygosity deficiency in the Hmong sample based on results from BOTTLENECK. The data from the EA, AA, Chinese and Thai samples do not violate the assumptions of mutation-drift equilibrium. These data suggest that the populations in this study have been large and at equilibrium for a relatively long period, with the exception of the Hmong population (as represented by the individuals we sampled). Unknown migrants or recent admixture can introduce new alleles into a population at initial low frequencies, mimicking the pattern caused by population expansion. Such recent admixture is not a likely explanation of the data in this case in light of successful clustering of the Hmong sample when analyzed with samples from the two populations most likely to contribute to hypothetical admixture: Thai and Chinese.

STRUCTURE, and other clustering algorithms, detect admixture and quantify population differentiation through differences in population allele frequencies. These differences which allow for successful clustering arise through various evolutionary forces and are shaped by ascertainment processes which must also be considered when identifying populations suitable for gene mapping studies or interpreting estimates of inter or intra-population genetic distance. The ability to differentiate between East Asian populations that have diverged recently relative to major continental populations indicates that it may also be possible to use more easily-accessible closely related populations, such as European, for admixture mapping if marker choice and population history are taken into account.

## Methods

### Populations and sampling

The Asian populations in this study were collected as part of an ongoing gene mapping study. Samples of self-identified Thai (N = 45) and Chinese (N = 29) were obtained from a blood drive in Bangkok, Thailand. The Thai and Chinese samples used in this study were selected to include only subjects for whom all four grandparents were reported to have the same self-identified ethnicity as the subject. The Hmong, a Miao-Yao-speaking group of the Austro-Thai language family, are an endogamous tribal population with an estimated total population throughout China, Laos, Vietnam, and Burma of eight million, approximately 120,000 of whom reside in Thailand. Chinese written history documents the presence of Hmong in Central China at least 2,300 years ago and their migration to Southern China several hundred years later. Migrations farther south have occurred since the seventeenth century [47]. Hmong refugees fleeing military conflict in Laos have periodically been resettled since 1975 in the U.S., France, and Australia. Hmong samples (N = 103) were obtained in two Hmong villages in northern Thailand. Data on grandparents' reported ethnic affiliation were not available for the Hmong subjects. The dataset also included samples of unrelated African Americans (AA, N = 54) and European Americans (EA, N = 91), a subset of a sample described elsewhere [15]. Both EA and AA samples were self-identified as such, and these identifications were previously confirmed via Bayesian marker clustering [15]. After immediate relatives were discovered and excluded from analysis (see below), sample sizes were reduced as follows: Hmong (N = 70) and Chinese (N = 28). No close relative pairs were found within the remaining three population samples. All subjects provided informed consent as approved by the appropriate institutional review boards.

### Markers and genotyping

For the three East Asian populations, DNA was extracted directly from blood using PaxGene materials and the manufacturer's specified protocol (Qiagen, Valencia CA, USA) (Hmong) or standard phenol/chloroform methods (Thai and Chinese). All samples were genotyped for thirty-two unlinked autosomal STR markers. The panel is comprised of the 15 tetranucleotides in the AmpF/STR Identifiler PCR Amplification kit (PE Applied Biosystems, Foster City, CA, USA) (D8S1179 [GenBank:AX412206], D21S11 [GenBank:AJ550387], D7S820 [GenBank:NC_000007], CSF1PO [GenBank:AF076965], D3S1358 [UniSTS:148226], TH01 [UniSTS:240639], D13S317 [GenBank:G09017], D16S539 [GenBank:AF249681, D2S1338 [GenBank:G08202], D19S433 [GenBank:G08036 ], vWA [UniSTS:240641], TPOX [GenBank:M25706], D18S51 [GenBank:L18333 ], D5S818 [GenBank:G08446 and FGA [GenBank:G3347]) and an additional 17 dinucleotide repeats (D17S799 [GenBank:Z16830], D8S272 [GenBank:Z17250], D7S640 [GenBank:Z23671], D8S1827 [GenBank:Z50970], D22S274 [GenBank:Z16730, D5S407 [GenBank:Z16723], D2S162 [GenBank:Z17035], D10S197 [GenBank:Z16611], D11S935 [GenBank:Z17148], D9S175 [GenBank:Z17021], D5S410 [GenBank:Z16825], D7S2469 [GenBank:Z53000], D16S3017 [GenBank:Z52036], D10S1786 [GenBank:Z51854], D15S1002 [GenBank:Z53249], D6S1610 [GenBank:Z53131], and D1S2628 [GenBank:Z52173]). The amelogenin locus, included in the AmpF/STR Identifiler PCR Amplification kit for sex identification, was not included in any analyses. All STR markers were analyzed on an ABI PRISM 3100 semiautomated capillary fluorescence sequencer. Data were scored using Genemapper (ABI). We have previously used this marker panel to determine and statistically correct for ancestry in case-control studies and genome-wide linkage studies [1,2,5,8].

### Statistical analyses

#### Population differentiation

Because variance of STRUCTURE results increases with small sample sizes [15], each run was repeated five times. However, results did not vary notably for each of the five runs given a set of conditions. For analysis of the three East Asian populations alone, the parameters used were K = 2 and K = 3, 50,000 burn-in and 50,000 Markov chain Monte Carlo (MCMC) iterations. For analysis of all five populations in this study, the parameters used were K = 2, K = 3, K = 4, and K = 5, with 50,000 burn-in and 50,000 MCMC iterations. These STRUCTURE runs were each carried out with all 32 markers and then with the 15 tetranucleotide markers and the 17 dinucleotide markers separated into two marker panels. The posterior probability for each value of "K" was calculated to determine the "K" that best fit the data for each set of populations and markers. The self-reported population of origin was not used as additional data by STRUCTURE and the presence of admixture was assumed.

#### Effective population size

The program BOTTLENECK evaluates populations for evidence of a recent rapid change in effective population size, according to differences between Nei's gene diversity, or unbiased expected heterozygosity (H_z_) based on observed allele frequencies versus expected equilibrium gene diversity (H_eq_), simulated based upon an assumed mutation model, number of alleles, and number of gene copies (2N for a diploid system) for each locus. Based on simulations of a coalescent process in which observed alleles at a locus are traced back to a hypothetical common ancestral allele, BOTTLENECK predicts present-day allele frequencies assuming constant population size. This results in a H_eq _value for the present-day population. Significant deviations from this predicted value are used to infer drastic changes in effective population size which have occurred in the recent past. A significant heterozygosity excess (H_z _> H_eq_) indicates a possible bottleneck while a significant deficit (H_z _< H_eq_) indicates a possible expansion. Observed heterozygosity is the percentage of heterozygous individuals in a sample for a locus and is based on observed genotypes while (H_z_) is the probability that two alleles chosen at random from the population sample will not be identical, correcting for sample size, and is an indirect measure of the extent to which allele frequencies for a locus are evenly distributed.

Significance of deviations from H_eq _was tested under the two-phased model of mutation (TPM) which assumes that the majority of mutations are single step mutations, as in the stepwise mutation model but allows for some multi-step mutations, which are more likely to be observed in dinucleotide repeats and may be a more accurate model for microsatellite mutation than the SMM [48]. BOTTLENECK allows the user to specify the percent of multi-step mutations assumed and the variance of allele size for the mutation model. BOTTLENECK authors suggest a percent of multi-step mutations between 5 and 10. It has been shown that incidence of type I error (detecting a bottleneck when a population has been at equilibrium) for the algorithm used in BOTTLENECK increases when assumed parameters are overestimated [49]. Therefore, based on detection of predicted bottlenecks for the AA, Chinese and EA populations when larger values were used, variance was set conservatively at 20 and percent of multi-step mutations was set at 5. The number of iterations of the simulated coalescent process under the TPM was 1000.

#### Relatedness

We used marker genotypes to identify, and then exclude from the analysis sample, closely related subjects who may not have identified themselves as such. The admixture model in STRUCTURE assumes HWE and linkage equilibrium within subpopulations; the use of close relatives within a sample would violate those assumptions and possibly result in false cluster detection [33]. Similarly, BOTTLENECK software assumes no close relatives in a population sample [34]. Although potential subjects may be instructed that multiple family members should not participate, cultural differences in kin definitions, lack of understanding of instructions, or financial compensation of subjects may result in individuals disregarding such instructions. Maximum likelihood estimates of pairwise relationships (parent-offspring, full sib, half-sib, or unrelated) were produced using the program ML-Relate [50] for all possible pairs within each population. ML-Relate does not require pedigree information and therefore can be applied to a large anonymous sample.

#### Hardy Weinberg Equilibrium (HWE)

Tests for deviation from Hardy-Weinberg equilibrium were conducted for each locus within each population using the exact test for HWE based on a Markov chain method implemented in the web-based version of GENEPOP [51]. The parameters used were 5000 dememorizations, 500 batches, and 5000 iterations per batch. The parameter values were increased from defaults until the observed standard error for p-values was less than 0.01.

#### Heterozygosity

Allele frequencies, observed heterozygosity (H_o_) values, and Nei's gene diversity (H_z_) for each locus were calculated using MStools [52]. For a diploid system, H_z _is calculated as H_z _= 2N(1-∑pi^2^)/2N -1, where N is the number of individuals sampled, and pi is the frequency of the i^th ^allele [53].

#### Marker information content

Markers were evaluated for delta (δ) [54], a measure of marker information content, reflecting the ability of a marker to statistically differentiate between populations. To arrive at δ, the absolute values of allelewise frequency differences between two populations are added and this sum is divided in half, δ=12∑i=1L|piA−piB|
 MathType@MTEF@5@5@+=feaafiart1ev1aaatCvAUfKttLearuWrP9MDH5MBPbIqV92AaeXatLxBI9gBaebbnrfifHhDYfgasaacH8akY=wiFfYdH8Gipec8Eeeu0xXdbba9frFj0=OqFfea0dXdd9vqai=hGuQ8kuc9pgc9s8qqaq=dirpe0xb9q8qiLsFr0=vr0=vr0dc8meaabaqaciaacaGaaeqabaqabeGadaaakeaaiiGacqWF0oazcqGH9aqpdaWcaaqaaiabigdaXaqaaiabikdaYaaadaaeWbqaamaaemaabaGaemiCaa3aa0baaSqaaiabdMgaPbqaaiabdgeabbaakiabgkHiTiabdchaWnaaDaaaleaacqWGPbqAaeaacqWGcbGqaaaakiaawEa7caGLiWoaaSqaaiabdMgaPjabg2da9iabigdaXaqaaiabdYeambqdcqGHris5aaaa@4421@ where piA
 MathType@MTEF@5@5@+=feaafiart1ev1aaatCvAUfKttLearuWrP9MDH5MBPbIqV92AaeXatLxBI9gBaebbnrfifHhDYfgasaacH8akY=wiFfYdH8Gipec8Eeeu0xXdbba9frFj0=OqFfea0dXdd9vqai=hGuQ8kuc9pgc9s8qqaq=dirpe0xb9q8qiLsFr0=vr0=vr0dc8meaabaqaciaacaGaaeqabaqabeGadaaakeaacqWGWbaCdaqhaaWcbaGaemyAaKgabaGaemyqaeeaaaaa@30A8@ and piB
 MathType@MTEF@5@5@+=feaafiart1ev1aaatCvAUfKttLearuWrP9MDH5MBPbIqV92AaeXatLxBI9gBaebbnrfifHhDYfgasaacH8akY=wiFfYdH8Gipec8Eeeu0xXdbba9frFj0=OqFfea0dXdd9vqai=hGuQ8kuc9pgc9s8qqaq=dirpe0xb9q8qiLsFr0=vr0=vr0dc8meaabaqaciaacaGaaeqabaqabeGadaaakeaacqWGWbaCdaqhaaWcbaGaemyAaKgabaGaemOqaieaaaaa@30AA@ are the allele frequencies for the *i*^th ^allele in population A and B. The more effective the marker is at differentiating between populations, the higher the value for δ [15]. In comparison to F_ST_, the measure δ is easily calculated and independent of mutation model assumptions.

## Authors' contributions

JBL designed the study, carried out statistical analyses and drafted the manuscript. BZY participated in the design and execution of statistical analyses. AS, NT, ST, AM, and KS participated in sample collection in Thailand. HRK carried out sample collection in the United States. RLR and TRD assisted in the writing of the manuscript and TRD is JBL's advisor. RTM participated in project coordination and sample collection, and the writing of the manuscript. JG participated in study design and supervision, project coordination, sample collection, and the writing of the manuscript. All authors read and approved the final manuscript.

## Supplementary Material

Additional file 1 Results from Fisher's test for deviation from HWE for each marker. The data provided represent, for each marker, for each population, the probability of the observed sample given the conditions for HWE are met and the standard error of the probability. Click here for file

Additional file 2 δ (delta) for each marker, for each population combination. The data provided show, for each marker, the values for δ (delta), a measure of marker informativeness between each pair of populations. Click here for file
