# Supplementary material for: Demographic changes and marker properties affect detection of human population differentiation
Source: BMC Genet. 2007 May 11;8:21. doi: 10.1186/1471-2156-8-21 (PMC1876243; doi:10.1186/1471-2156-8-21)
Supplement: Additional file 1 — Results from Fisher's test for deviation from HWE for each marker. The data provided represent, for each marker, for each population, the probability of the observed sample given the conditions for HWE are met and the standard error of the probability. [file 1471-2156-8-21-S1.doc]

| **Additional File 1.** Results from Fisher's test for deviation from HWE for each marker | | | | | | | | | | |
| --- | --- | --- | --- | --- | --- | --- | --- | --- | --- | --- |
|  | **EA** | | **AA** | | **Thai** | | **Chinese** | | **Hmong** | |
|  | p | SE | p | SE | p | SE | p | SE | p | SE |
| **Tetranucleotide** | | | | | | | | | | |
| CSF1PO | 0.076 | 0.002 | 0.758 | 0.002 | 0.497 | 0.002 | 0.053 | 0.001 | 0.188 | 0.003 |
| D2S1338 | 0.782 | 0.003 | 0.150 | 0.003 | 0.250 | 0.004 | 0.590 | 0.004 | 0.544 | 0.006 |
| D3S1358 | 0.562 | 0.004 | 0.864 | 0.001 | 0.868 | 0.002 | 0.134 | 0.002 | 0.182 | 0.003 |
| D5S818 | 0.889 | 0.002 | 0.941 | 0.001 | 0.374 | 0.004 | 0.094 | 0.002 | 0.525 | 0.003 |
| D7S820 | 0.205 | 0.003 | 0.730 | 0.002 | 0.672 | 0.003 | 0.403 | 0.002 | 0.594 | 0.004 |
| D8S1179 | 0.125 | 0.003 | 0.276 | 0.003 | ***0.048*** | 0.001 | 0.864 | 0.002 | 0.796 | 0.002 |
| D13S317 | ***0.027*** | 0.001 | 0.752 | 0.002 | 0.525 | 0.003 | 0.955 | 0.001 | 0.674 | 0.005 |
| D16S539 | 0.541 | 0.004 | 0.533 | 0.003 | 0.161 | 0.002 | 0.614 | 0.003 | 0.696 | 0.002 |
| D18S51 | 0.495 | 0.007 | 0.870 | 0.004 | 0.716 | 0.007 | 0.401 | 0.005 | 0.433 | 0.007 |
| D19S433 | 0.514 | 0.007 | 0.322 | 0.006 | 0.548 | 0.007 | 0.500 | 0.004 | 0.557 | 0.005 |
| D21S11 | 0.268 | 0.004 | 0.421 | 0.006 | 0.850 | 0.003 | 0.399 | 0.004 | 0.553 | 0.006 |
| FGA | 0.902 | 0.003 | 0.649 | 0.005 | 0.451 | 0.005 | 0.869 | 0.002 | 0.632 | 0.007 |
| TH01 | 0.234 | 0.002 | 0.164 | 0.002 | 0.586 | 0.003 | 0.405 | 0.003 | 0.679 | 0.003 |
| TPOX | 0.699 | 0.002 | 0.922 | 0.001 | 0.239 | 0.002 | 0.240 | 0.002 | 0.440 | 0.003 |
| vWA | 0.370 | 0.003 | 0.362 | 0.004 | 0.382 | 0.003 | 0.988 | 0.000 | 0.965 | 0.001 |
| **Dinucleotide** | | | | | |  | | | | |
| D17S799 | 0.912 | 0.003 | 0.291 | 0.004 | 0.796 | 0.003 | 0.625 | 0.003 | 0.944 | 0.001 |
| D8S272 | 0.113 | 0.003 | 0.612 | 0.006 | 0.650 | 0.005 | 0.160 | 0.004 | 0.737 | 0.002 |
| D7S640 | 0.390 | 0.008 | 0.138 | 0.005 | 0.301 | 0.004 | 0.823 | 0.005 | 0.464 | 0.010 |
| D8S1827 | 0.290 | 0.003 | 0.117 | 0.002 | 0.201 | 0.002 | 0.430 | 0.002 | 0.800 | 0.003 |
| D22S274 | 0.261 | 0.004 | 0.373 | 0.004 | 0.490 | 0.004 | 0.302 | 0.003 | 0.735 | 0.003 |
| D5S407 | 0.848 | 0.004 | 0.565 | 0.007 | 0.628 | 0.006 | 0.834 | 0.003 | 0.731 | 0.004 |
| D2S162 | 0.450 | 0.006 | 0.529 | 0.007 | 0.441 | 0.005 | 0.787 | 0.004 | 0.856 | 0.004 |
| D10S197 | 0.719 | 0.004 | 0.906 | 0.002 | 0.379 | 0.003 | 0.313 | 0.002 | 0.567 | 0.004 |
| D11S935 | ***0.028*** | 0.001 | 0.968 | 0.001 | 0.939 | 0.001 | 0.828 | 0.003 | 0.681 | 0.003 |
| D9S175 | 0.417 | 0.009 | 0.353 | 0.007 | 0.449 | 0.012 | 0.307 | 0.010 | 0.449 | 0.012 |
| D5S410 | ***0.033*** | 0.002 | 0.080 | 0.003 | 0.835 | 0.001 | 0.827 | 0.001 | 0.697 | 0.002 |
| D7S2469 | 0.568 | 0.010 | 0.891 | 0.003 | 1.000 | 0.000 | 1.000 | 0.000 | 0.607 | 0.009 |
| D16S3017 | 0.210 | 0.005 | 0.831 | 0.003 | ***0.049*** | 0.003 | 0.166 | 0.002 | 0.097 | 0.001 |
| D10S1786 | ***0.030*** | 0.001 | 0.718 | 0.002 | 0.830 | 0.002 | ***0.032*** | 0.001 | 0.254 | 0.004 |
| D15S1002 | 0.350 | 0.006 | 0.104 | 0.004 | 0.607 | 0.004 | 0.644 | 0.004 | 0.567 | 0.005 |
| D6S1610 | 0.438 | 0.006 | 0.344 | 0.005 | 0.910 | 0.001 | 0.834 | 0.002 | 0.745 | 0.003 |
| D1S2628 | 0.305 | 0.003 | 0.492 | 0.002 | 0.583 | 0.003 | 0.071 | 0.001 | 0.195 | 0.001 |
|  |  |  |  |  |  |  |  |  |  |  |

Four markers showed significant deviation from HWE in the EA sample. The Thai and Chinese samples showed significant deviation from HWE in one and two markers, respectively. Significant p-values are italicized and in bold. When a Bonferonni correction is made for multiple testing, none of these p values are significant.
