# Supplementary material for: Demographic changes and marker properties affect detection of human population differentiation
Source: BMC Genet. 2007 May 11;8:21. doi: 10.1186/1471-2156-8-21 (PMC1876243; doi:10.1186/1471-2156-8-21)
Supplement: Additional file 2 — δ (delta) for each marker, for each population combination. The data provided show, for each marker, the values for δ (delta), a measure of marker informativeness between each pair of populations. [file 1471-2156-8-21-S2.doc]

| **Additional File 2.** δ (delta) for each marker, for each population combination | | | | | | | | | | | | |
| --- | --- | --- | --- | --- | --- | --- | --- | --- | --- | --- | --- | --- |
|  | **Hmong/**  **EA** | | **Hmong/**  **AA** | **Hmong/**  **Thai** | **Hmong/**  **Chinese** | **EA/**  **AA** | **EA/**  **Thai** | **EA/**  **Chinese** | | **AA/**  **Thai** | **AA/**  **Chinese** | **Thai/**  **Chinese** |
| **Tetranucleotide** | | | | | | | | | | | | |
| **CSF1PO** | | 0.298 | 0.319 | 0.220 | 0.280 | 0.155 | 0.165 | 0.076 | | 0.219 | 0.155 | 0.108 |
| **D2S1338** | | 0.416 | 0.248 | 0.257 | 0.290 | 0.278 | 0.299 | 0.345 | | 0.191 | 0.291 | 0.215 |
| **D3S1358** | | 0.240 | 0.193 | 0.267 | 0.096 | 0.208 | 0.232 | 0.170 | | 0.150 | 0.153 | 0.207 |
| **D5S818** | | 0.347 | 0.394 | 0.136 | 0.248 | 0.186 | 0.256 | 0.203 | | 0.370 | 0.352 | 0.131 |
| **D7S820** | | 0.353 | 0.398 | 0.297 | 0.280 | 0.127 | 0.205 | 0.190 | | 0.273 | 0.291 | 0.114 |
| **D8S1179** | | 0.409 | 0.488 | 0.166 | 0.379 | 0.308 | 0.289 | 0.317 | | 0.358 | 0.154 | 0.245 |
| **D13S317** | | 0.604 | 0.778 | 0.232 | 0.364 | 0.229 | 0.403 | 0.370 | | 0.585 | 0.552 | 0.175 |
| **D16S539** | | 0.320 | 0.275 | 0.186 | 0.239 | 0.225 | 0.155 | 0.119 | | 0.229 | 0.235 | 0.083 |
| **D18S51** | | 0.342 | 0.435 | 0.222 | 0.189 | 0.245 | 0.287 | 0.193 | | 0.419 | 0.277 | 0.184 |
| **D19S433** | | 0.369 | 0.334 | 0.256 | 0.249 | 0.246 | 0.332 | 0.338 | | 0.332 | 0.273 | 0.233 |
| **D21S11** | | 0.292 | 0.475 | 0.301 | 0.297 | 0.210 | 0.269 | 0.303 | | 0.354 | 0.341 | 0.138 |
| **FGA** | | 0.456 | 0.388 | 0.482 | 0.383 | 0.275 | 0.149 | 0.241 | | 0.311 | 0.176 | 0.245 |
| **TH01** | | 0.338 | 0.314 | 0.201 | 0.198 | 0.234 | 0.436 | 0.466 | | 0.289 | 0.351 | 0.156 |
| **TPOX** | | 0.235 | 0.315 | 0.331 | 0.229 | 0.290 | 0.153 | 0.069 | | 0.335 | 0.358 | 0.187 |
| **vWA** | | 0.268 | 0.514 | 0.196 | 0.123 | 0.356 | 0.194 | 0.201 | | 0.463 | 0.456 | 0.102 |
| **Dinucleotide** | | | | | | |  | | | | | |
| **D17S799** | 0.446 | | 0.566 | 0.157 | 0.162 | 0.513 | 0.510 | 0.350 | 0.588 | | 0.640 | 0.181 |
| **D8S272** | 0.306 | | 0.421 | 0.117 | 0.164 | 0.346 | 0.311 | 0.295 | 0.423 | | 0.377 | 0.203 |
| **D7S640** | 0.460 | | 0.517 | 0.572 | 0.379 | 0.352 | 0.309 | 0.294 | 0.424 | | 0.291 | 0.362 |
| **D8S1827** | 0.258 | | 0.517 | 0.036 | 0.239 | 0.391 | 0.240 | 0.101 | 0.499 | | 0.430 | 0.214 |
| **D22S274** | 0.261 | | 0.220 | 0.329 | 0.293 | 0.207 | 0.299 | 0.433 | 0.413 | | 0.463 | 0.183 |
| **D5S407** | 0.226 | | 0.347 | 0.215 | 0.190 | 0.328 | 0.183 | 0.255 | 0.427 | | 0.393 | 0.258 |
| **D2S162** | 0.425 | | 0.477 | 0.284 | 0.275 | 0.458 | 0.228 | 0.259 | 0.412 | | 0.441 | 0.177 |
| **D10S197** | 0.271 | | 0.348 | 0.290 | 0.264 | 0.233 | 0.182 | 0.244 | 0.286 | | 0.248 | 0.156 |
| **D11S935** | 0.378 | | 0.509 | 0.304 | 0.311 | 0.578 | 0.326 | 0.322 | 0.573 | | 0.552 | 0.258 |
| **D9S175** | 0.323 | | 0.607 | 0.250 | 0.138 | 0.486 | 0.277 | 0.287 | 0.584 | | 0.597 | 0.224 |
| **D5S410** | 0.422 | | 0.556 | 0.132 | 0.128 | 0.443 | 0.437 | 0.376 | 0.522 | | 0.569 | 0.207 |
| **D7S2469** | 0.291 | | 0.517 | 0.242 | 0.260 | 0.333 | 0.289 | 0.373 | 0.490 | | 0.548 | 0.100 |
| **D16S3017** | 0.397 | | 0.473 | 0.154 | 0.220 | 0.291 | 0.375 | 0.340 | 0.428 | | 0.450 | 0.136 |
| **D10S1786** | 0.462 | | 0.393 | 0.262 | 0.343 | 0.484 | 0.450 | 0.516 | 0.463 | | 0.415 | 0.218 |
| **D15S1002** | 0.634 | | 0.558 | 0.256 | 0.326 | 0.479 | 0.420 | 0.352 | 0.389 | | 0.422 | 0.143 |
| **D6S1610** | 0.369 | | 0.346 | 0.230 | 0.264 | 0.233 | 0.301 | 0.344 | 0.237 | | 0.234 | 0.183 |
| **D1S2628** | 0.364 | | 0.479 | 0.238 | 0.214 | 0.452 | 0.352 | 0.393 | 0.517 | | 0.654 | 0.221 |
| **Average** |  | |  |  |  |  |  |  |  | |  |  |
| **32 markers** | 0.362 | | 0.428 | 0.244 | 0.250 | 0.318 | 0.291 | 0.285 | 0.392 | | 0.379 | 0.186 |
| **Tetranucleotide** | 0.352 | | 0.391 | 0.250 | 0.256 | 0.238 | 0.255 | 0.240 | 0.325 | | 0.294 | 0.168 |
| **Dinucleotide** | 0.370 | | 0.462 | 0.239 | 0.245 | 0.388 | 0.323 | 0.325 | 0.451 | | 0.454 | 0.201 |

Delta (**δ)** values are shown for each marker for each population combination. Also shown are the average delta values over all 32 markers, for tetranucleotide markers alone, and for dinucleotide markers alone for each population combination.
